# Pathling: analytics on FHIR

**DOI:** 10.1186/s13326-022-00277-1

**Published:** 2022-09-08

**Authors:** John Grimes, Piotr Szul, Alejandro Metke-Jimenez, Michael Lawley, Kylynn Loi

**Affiliations:** Australian e-Health Research Centre, CSIRO, Level 7, Surgical Treatment and Rehabilitation Service (STARS), Royal Brisbane and Women’s Hospital, Herston, 4029 Queensland Australia

**Keywords:** Data analytics, Interoperability, Clinical terminology, FHIR, FHIRPath, SNOMED CT

## Abstract

**Background:**

Health data analytics is an area that is facing rapid change due to the acceleration of digitization of the health sector, and the changing landscape of health data and clinical terminology standards. Our research has identified a need for improved tooling to support analytics users in the task of analyzing Fast Healthcare Interoperability Resources (FHIR^®^) data and associated clinical terminology.

**Results:**

A server implementation was developed, featuring a FHIR API with new operations designed to support exploratory data analysis (EDA), advanced patient cohort selection and data preparation tasks. Integration with a FHIR Terminology Service is also supported, allowing users to incorporate knowledge from rich terminologies such as SNOMED CT within their queries. A prototype user interface for EDA was developed, along with visualizations in support of a health data analysis project.

**Conclusions:**

Experience with applying this technology within research projects and towards the development of analytics-enabled applications provides a preliminary indication that the FHIR Analytics API pattern implemented by Pathling is a valuable abstraction for data scientists and software developers within the health care domain. Pathling contributes towards the value proposition for the use of FHIR within health data analytics, and assists with the use of complex clinical terminologies in that context.

**Supplementary Information:**

The online version contains supplementary material available at 10.1186/s13326-022-00277-1.

## Background

Fast Healthcare Interoperability Resources (FHIR^®^) is a health data standard that is experiencing rapid adoption within electronic health record (EHR) systems, driven by its ability to reduce the costs of interoperability and also its ability to enable a new ecosystem of third-party applications [1]. However, the adoption of FHIR within analytics and research remains low, as the standard is still young and the tooling support for FHIR data analytics is still relatively immature [2].

FHIR has catalyzed the rapid delivery of a large number of new applications that are capable of integrating with EHR data through the FHIR API, but the vast majority of these applications are limited to viewing data related to a single patient. One factor influencing this may be that the FHIR API is not currently well suited to queries that aggregate and categorize data across a population.

A parallel and related trend within health information systems is the investment in higher-quality structured data through coding of clinical records at the point of care. Increasingly, EHR implementations provide the ability for clinicians to add diagnoses, procedures, medications and other clinical concepts to medical records using advanced terminologies such as SNOMED CT [3]. This provides the potential for more detailed analysis through access to more specific clinical concepts, and also the ability to query the underlying ontology based upon additional attributes and relationships to other clinical concepts.

The level of skill and experience required to make the best use of FHIR and clinical terminology within a data analysis workflow is currently very high, and in short supply.

### User research

Fifty-three (53) interviews were conducted with forty-two (42) different users in various target customer segments to achieve a better understanding of workflow and unmet requirements. These interviews presented open-ended questions relating to current challenges within the area of health data analytics.

This process helped to validate our assumptions about the market, confirming that there is a group of early adopters that are interested in augmenting their workflow with new tools in this area. It also identified a range of different analytic use cases that we could assess in terms of priority and fit with our product.

Based on what we learned from this process, we selected the following three primary use cases to serve as the focus of our work:

1. **Exploratory data analysis** - Exploration of hypotheses, assessment of assumptions, and selection of appropriate statistical tools and techniques [4].
2. **Patient cohort selection** - Selection and retrieval of patient records based upon complex inclusion and exclusion criteria.
3. **Data preparation** - Processing and re-shaping data in preparation for use with statistical and machine learning tools.

### Challenges

FHIR is complex, highly nested and graph-like in nature. This makes its representation within traditional relational data models challenging, especially when simplifying query logic is a key goal. Performance can also be an issue when designing data structures optimized for analytic query across a general set of use cases.

Clinical terminology can also be a particularly difficult type of reference data to work with, especially within the context of designing a data repository optimized for analytics. Complex clinical terminologies such as SNOMED CT are very large in terms of number of concepts, and the size of their query-optimized representation within a database can become unbounded if you take into account that they can be composed and queried in arbitrary ways, using expressions [5]. Simplifying the representation of clinical terminology at the cost of query expressivity is at odds with our goal of maximizing the clinical utility of the tools we build.

To maximize the impact of the use of this technology within health care delivery, the solution needs to be practical and fit for use within the context of a large health care organization or jurisdiction. Specifically, the solution needs to be scalable in terms of data volume, enabling efficient query execution across data sets that could include longitudinal records for tens of millions of patients. The design also needs to enable real-time processing use cases, which are increasingly important with the deployment of a growing number of connected medical devices [6].

### Prior research

There have been numerous efforts to understand this problem space, and these initiatives have been a major influence upon the work described in this paper.

The Observational Health Data Sciences and Informatics (OHDSI) program has produced a large volume of work in the area of health data analytics, including the creation of the Observational Medical Outcomes Partnership (OMOP) Common Data Model (CDM) [7]. OMOP provides a target data model for health data analytics, along with common vocabularies and analytic routines that can be run over the common model. OMOP has a rich ecosystem of tools that have been developed to aid in its use and implementation. An example of this is ATLAS [8], a user interface designed to facilitate analytic query over the OMOP data model. Pathling aims to provide functionality that enables the development of similar tools within the FHIR ecosystem.

Use of the OpenEHR model within health data analytics has also been the subject of prior research. Notably, Ma et al. [9] described a query language that went on to become the basis of the Archetype Query Language (AQL) [10], a standard way of querying data from OpenEHR-based systems. AQL has since gone on to be implemented within a number of EHR and analytic software products, and provides clues as to the required design features of this type of capability.

Liu et al. [11] examined a number of different ways to make bulk FHIR data available for analytic query, and concluded that Apache Parquet [12] is the best candidate for storage and query of FHIR data in the context of large-scale analytics using Apache Spark. Our work also confirmed these findings, with Parquet providing good performance and also offering good compatibility with other tools and libraries that are used within health data analytics and machine learning.

The Cerner Corporation produced a library called Bunsen [13] that encodes FHIR resources within Apache Spark [14] data sets, and by extension, Apache Parquet. This foundational work has made it easier to load, transform and analyze FHIR data at scale, and some of this code has formed the foundation of the implementation described in this paper. Cerner has also been involved with the Structured Query Language (SQL) on FHIR proposal [15], which is a projection of the FHIR data model on to the SQL language and relational query model. The Parquet encoding pattern used in Bunsen and Pathling is based upon this proposal.

Google has described and implemented a method for encoding FHIR data using Protocol Buffers [16]. Google has also shared many tools and techniques [17] for using FHIR with the BigQuery analytics platform, integrating with the FHIR Bulk Data API and using FHIR data within cloud-based data processing and machine learning pipelines.

The A3 project [18], created as part of the Sync for Science collaboration, provides an example of how FHIR data can be normalized in service of simplifying the task of authoring analytic queries, and also demonstrates user interface patterns that can effectively support the authoring of cohort criteria.

## Implementation

**Fig. 1:**
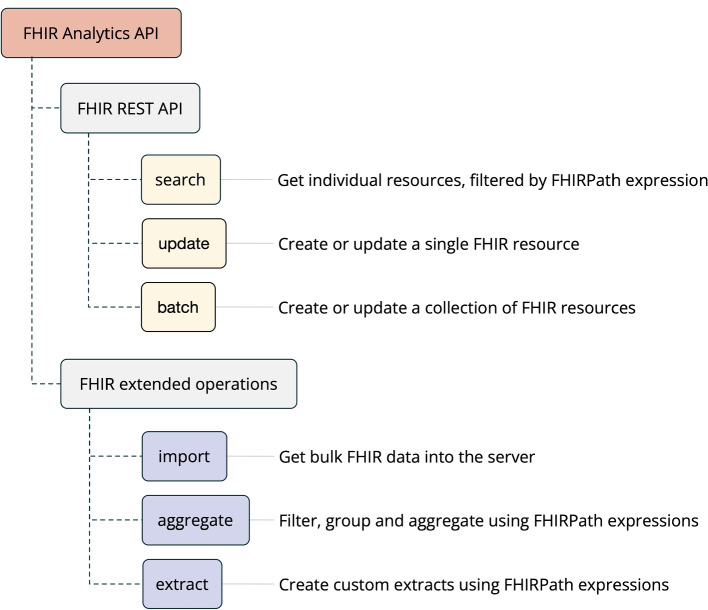
Operations of a FHIR Analytics API

We addressed the identified use cases through the design of a “FHIR Analytics API” (see Fig. 1), which refers to a specialization of the FHIR API that focuses on providing functionality useful for health data analytics applications. FHIR provides standard mechanisms for extending the functionality of its API such as extended operations and search profiles, and we have taken advantage of these to deliver new functionality that can be consumed by existing FHIR client software in a frictionless way.

The additional operations purposefully target capabilities that are not currently possible or not easily achieved using the core FHIR REST API specification, such as aggregation and transformation of data. Search capabilities are also provided that allow for more expressive queries than the core FHIR Search API. The FHIR Analytics API is designed to complement, rather than replace, the transactional capabilities of the standard FHIR REST API.

### Import operation

**Fig. 2:**
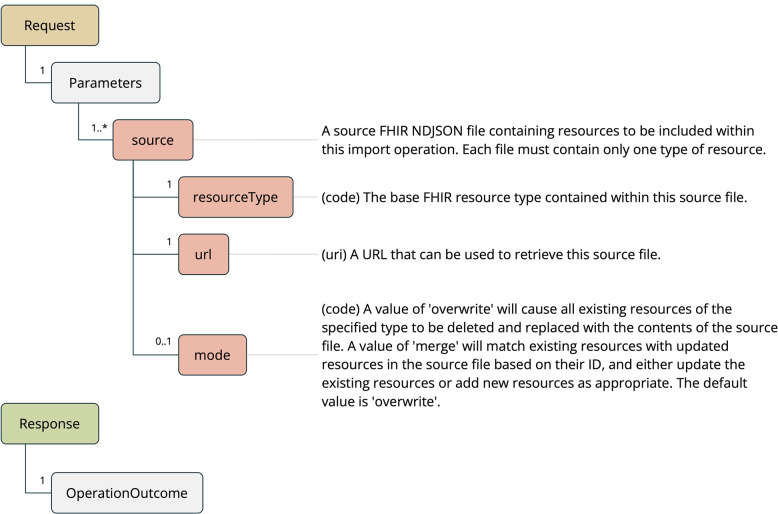
Import operation

The first feature provided by the FHIR Analytics API is the Import Operation (see Fig. 2), which provides a way of making bulk data available to the server and preparing it for subsequent queries. A standard FHIR server can import data using the create and update REST operations (and batches of these operations), however this approach does not scale well to the volume of data needed for analytic applications.

The Import Operation is designed to accept data that has been extracted from systems through the HL7 FHIR Bulk Data Access interface [19]. This specification is achieving rapid implementation within vendor implementations [20], providing a standard way of sharing bulk data with other systems, including analytics tools. This operation is also based upon the Draft Bulk Import Implementation Guide [21], an effort by the FHIR community to design a standard operation for the efficient import of large data sets.

One of the differentiating aspects of a bulk data import request (relative to the FHIR transaction/batch operation) is the method for providing data to the server. Within FHIR transaction and batch requests, data is provided inline within the HTTP request itself. In a bulk import request, the client provides the server with URLs that the data files can be retrieved from. The protocols used within these URLs can include sources other than HTTP. Pathling currently supports the retrieval of data from Hadoop File System (HDFS) [22] and Amazon S3 URLs, which both use protocols that are optimized for the retrieval of large files.

The other difference with bulk import is that the response may be asynchronous, responding immediately with a URL rather than making the client wait for the completion of the operation. This URL can be used to receive updates on the progress of the operation and information about how to retrieve the final result. This is important when operations can take long periods of time to complete, and be otherwise hampered by timeouts and limitations on request size within HTTP implementations.

FHIR data are made available to the server via NDJSON [23], which is a way of representing collections of FHIR resources that is more bandwidth and storage efficient than a FHIR Bundle. Each NDJSON file provided to the operation contains a collection of instances of a single FHIR resource type.

### Aggregate operation

**Fig. 3:**
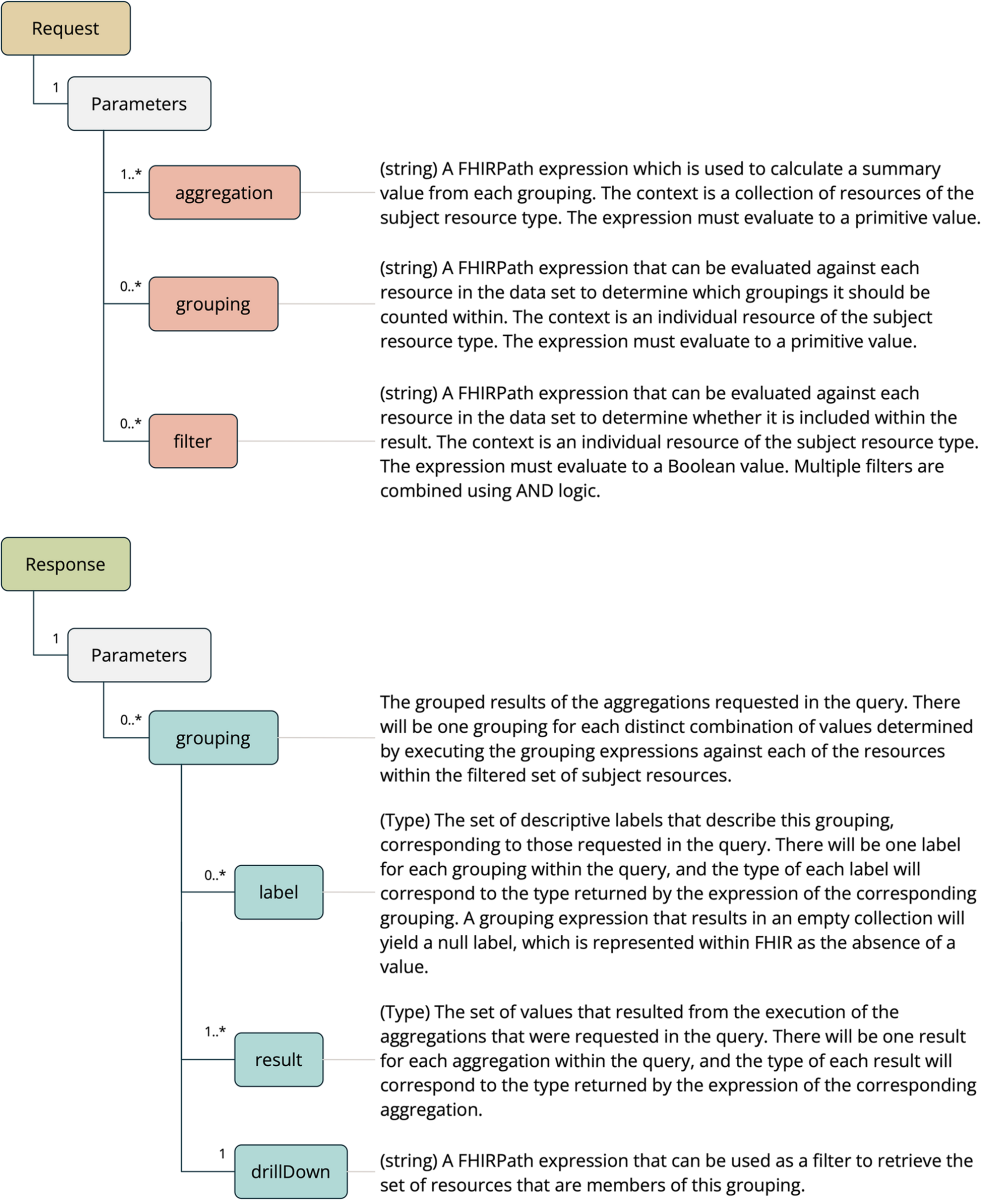
Aggregate operation

The second feature of the FHIR Analytics API is the Aggregate Operation (see Fig. 3), which facilitates the execution of aggregation-based queries across a data set. The concept of the aggregate operation is very similar to that of a “pivot table” [24], commonly used within spreadsheet applications. This provides a familiar and flexible set of tools that can power a range of different applications that satisfy the “Exploratory data analysis” use case, including visualizations.

Expressions are used to describe the aggregations, groupings and filters that form the definition of the query. We use FHIRPath [25] for this purpose, a language that is used within the FHIR specification. FHIRPath is a graph-based traversal language, and allows for “paths” through the resource graph to be described succinctly and in absence of some of the complexities of dealing with FHIR data types, cardinalities, resource references, and missing data.

The Aggregate Operation is defined within FHIR as a “type-level” operation, which means that it is invoked on a particular resource type, with the collection of all resources of that type becoming the subject of the operation. This subject resource becomes the root context for the FHIRPath expressions that are supplied to the operation.

One or more aggregation expressions can be provided, serving the purpose of defining a set of summary values that are to be calculated over the data set. Some examples of aggregation expressions are “count” and “sum”.

Grouping expressions are evaluated against each resource in order to determine the set of groups that it should be counted within. Aggregation expressions are then executed to determine a result for each group of resources, and these results are provided within the response to the operation.

Filter expressions serve to constrain the scope of the input collection. Resources that do not evaluate as true for all supplied filter expressions are excluded from the results.

A simple worked example of the Aggregate Operation follows, given the data set of patients, with a gender, deceased status (“deceasedBoolean”) and birth date (“birthDate”) in Table 1.

**Table 1:** Example Patient data set

id	gender	deceasedBoolean	birthDate
1	male	false	2013-06-10
2	female	true	1999-05-12
3	male	true	1955-07-08
4	female	true	1972-03-14
5	other	false	1981-03-27
6	male	false	1991-05-05

A query can be composed that contains one aggregation expression, A:
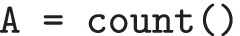


The query contains two grouping expressions: B, grouping on the patient gender, and; C, grouping on the deceased status of the patient:
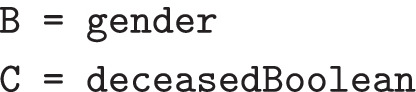


Finally, the query defines a single filter D, filtering out all patients with a birth date from January 1, 2000:
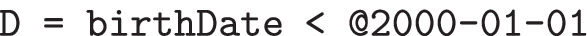


Table 2 shows the result, which contains a row for each distinct combination of B and C found in the data set, along with the result of A calculated over the resources that are a part of that grouping.

**Table 2:** Example result from Aggregate Operation

B	C	A
female	true	2
male	true	1
male	false	1
other	false	1

Note that patient 1 was omitted from the results by filter D. The combinations [female, false] and [other, true] were not included, as there were no resources matching these values.

### FHIRPath search profile

The third feature of the FHIR Analytics API is FHIRPath-powered search functionality, surfaced through a FHIR search profile. This search profile accepts any number of FHIRPath filter expressions, returning a Bundle resource containing matching resources within the server.

This search parameter can be used in conjunction with the Aggregate Operation, which returns a “drill down” expression with each grouping in the response. Each of these expressions can be used with the search API to retrieve the individual resources that comprise a grouping within the Aggregate Operation result. This provides the basis for delivering applications that satisfy the aforementioned “Patient cohort selection” use case.

FHIRPath filter expressions can also be combined with standard search parameters from the FHIR search specification. This allows the client to customize the representation of results in the response. An example of this is “_elements”, which controls the data elements that are included with each resource in the response.

An example drill-down expression for the [female, true] grouping in the previous example Aggregate response would be:
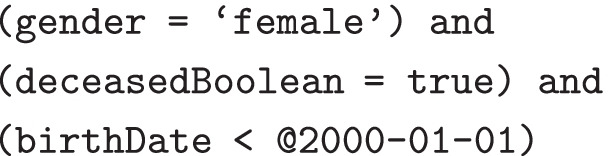


This would return patients 2 and 4 from the example data set. Note that this incorporates one of the distinct value pairs resulting from groupings B and C as a matching condition, as well as the overall filter condition from expression D.

FHIRPath provides additional expressive power relative to the FHIR Search API, such as:

1. The ability to refer to any element, not just those that are the subject of a defined search parameter;
2. Unlimited nesting and bracketing of expressions;
3. The ability to follow resource references that traverse multiple levels of relationships, and;
4. Support for complex terminology operations within criteria.

### Extract operation

**Fig. 4:**
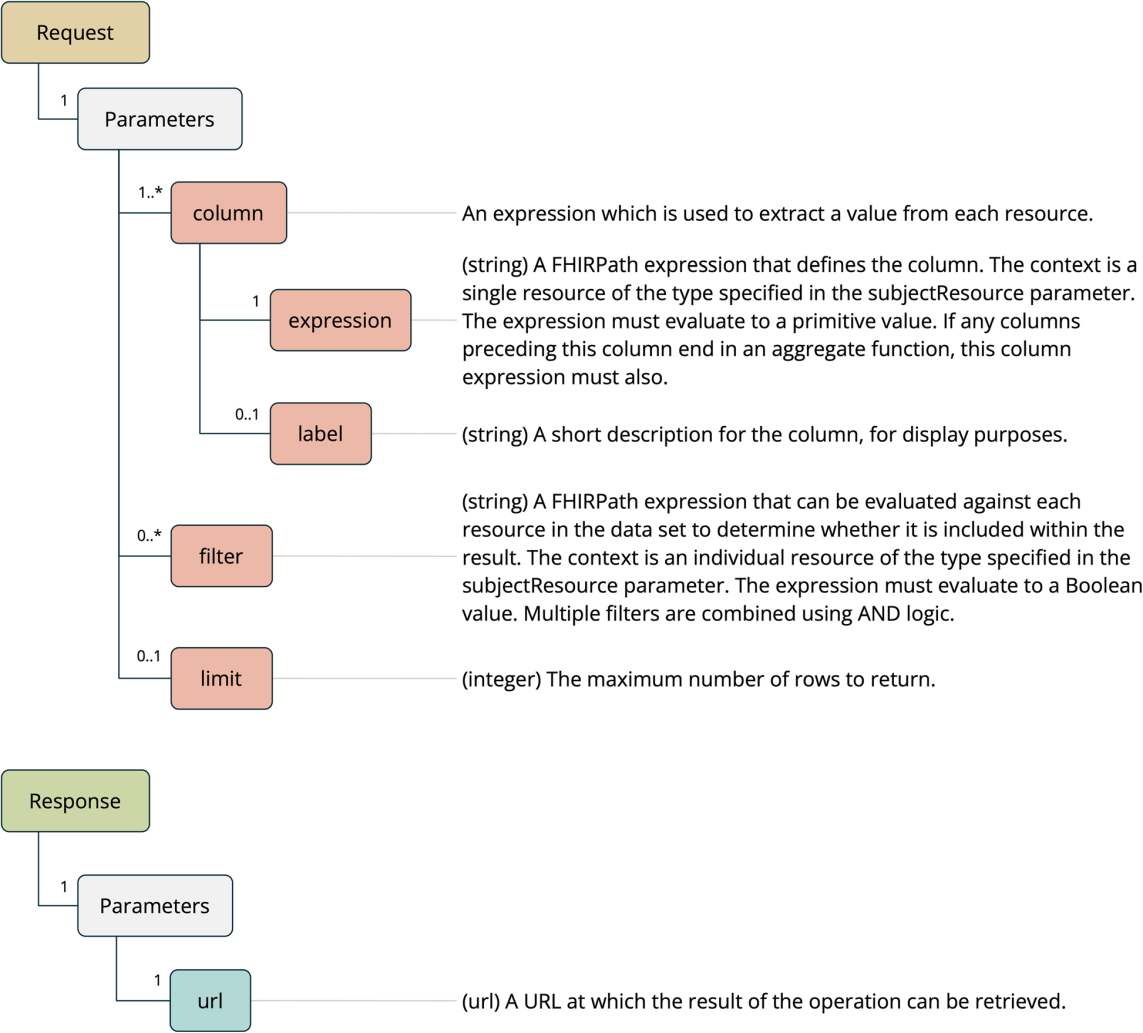
Extract operation

Finally, the Extract Operation (see Fig. 4) is designed to create custom data extracts for input into other tools and workflows. It is designed to simplify the task of reaching into the FHIR data model and producing a flattened rendition of selected parts of the data set. The full power of FHIRPath is available for use within this operation, including terminology functions.

The Extract Operation takes a subject resource type and a set of FHIRPath expressions as input. The result of the operation is a delimited text file with a column for each input expression. Each row in the file contains the result of the execution each of those expressions against each FHIR resource.

A simple worked example of the Extract Operation follows, using the previously stated example Patient data set in Table 1, a Practitioner data set in Table 3 and a MedicationRequest data set in Table 4.

**Table 3:** Example Practitioner data set

id	name	identifier
1	Ampelios Ajeet	[ { system: “urn:example:provider-id”, value: “ajeet” } ]
2	Leon Gautselin	[ { system: “urn:example:provider-id”, value: “gaut” } ]
3	Felicita Cyra	[ { system: “urn:example:provider-id”, value: “cyra” } ]

**Table 4:** Example MedicationRequest data set

id	medicationCodeableConcept	subject	requester
1	[ { text: “Atorvastatin” } ]	Patient/4	Practitioner/1
2	[ { text: “Levothyroxine” } ]	Patient/4	Practitioner/1
3	[ { text: “Lisinopril” } ]	Patient/1	Practitioner/3
4	[ { text: “Metformin” } ]	Patient/2	Practitioner/2

A query can be composed that uses MedicationRequest as the subject resource. It contains four column expressions: D, the resource ID of the MedicationRequest; E, the resource ID of the subject Patient; F, the provider identifier of the prescribing Practitioner, and; G, the text representation of the prescribed medication.
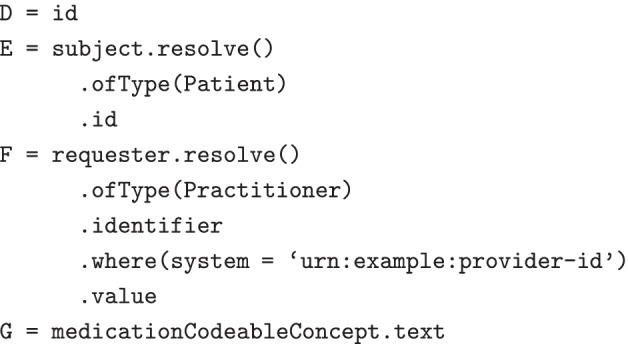


Table 5 shows the result, which contains a row for each MedicationRequest resource. Each row contains the result of evaluating the expressions D, E, F and G against the resource.

**Table 5:** Example result from Extract operation

D	E	F	G
1	4	ajeet	Atorvastatin
2	4	ajeet	Levothyroxine
3	1	cyra	Lisinopril
4	2	gaut	Metformin

The Extract operation returns a URL that can be used to retrieve the result of the operation, rather than returning the result inline within the HTTP response. This is to account for the potential large size of an Extract result when executed upon a data set with a large number of resources.

The Extract operation also features a “limit” parameter that allows the user to specify the maximum number of rows to be returned within the result. This can be useful to preview the format of a result without executing the query against the entire data set.

### Terminology functions

As an important source of meaning within the information model, terminology is a core element of both the FHIR and FHIRPath specification. The FHIRPath specification contains a number of terminology functions that we have implemented and extended to provide a set of capabilities useful for analytic query.

In our implementation, these functions delegate terminology queries to an implementation of the FHIR Terminology Service API. This approach removes the need to import and maintain a read-optimized view of terminology data, and it creates a clear separation of concerns between the query engine and the source of terminology knowledge.

#### Value set membership

Value set membership testing is fundamental to the task of categorizing and extracting ontological information from codes. We provide this capability through the implementation of the FHIRPath function, “memberOf”. This function takes a URI that identifies a defined set of codes, and uses the terminology server to determine the membership of a set of input concepts based on its knowledge of the code systems involved.

An example of the “memberOf” function follows, given the data set of SNOMED CT coded procedures in Table 6. This example uses a SNOMED CT implicit ValueSet URI that refers to an Expression Constraint Language (ECL) expression, which is a standard mechanism within FHIR for describing a set of SNOMED CT concepts.

**Table 6:** Example data set used with memberOf function

id	code
1	232850000 |Aortoventriculoplasty|
2	63377001 |Open core needle biopsy of liver|
3	428581004 |Percutaneous transluminal ablation of accessory pathway|
4	25321000 |Thoracoscopic pneumonectomy|

Note that the actual structure of the “code” field in the FHIR Procedure resource is CodeableConcept, which is a complex structure that can accommodate multiple codings along with text. This has been simplified for this example to a simple SNOMED CT code, along with a label for readability.

Given that a search query was made to the Procedure resource, with the following FHIRPath condition:
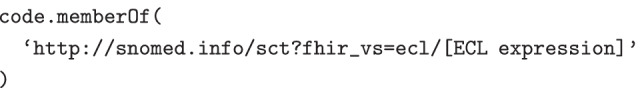


Where the following ECL expression was used within the argument:
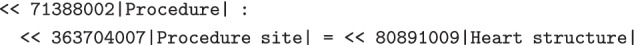


The result of this operation would be a FHIR Bundle containing the Procedure resources with SNOMED CT codes that are a type of “Procedure”, where the “Procedure site” is a “Heart structure”. There are 1,638 concepts that would match this query within the international release of SNOMED CT (February 2022), out of a total of 58,737 procedure concepts. The operation would return the Procedure resources with IDs 1 and 3, which are both types of procedures that are performed on the heart.

The information required to derive this result includes the subsumption relationships and attributes of the codes that exist within SNOMED CT itself. The terminology server has knowledge of these relationships along with the ability to understand ECL, and Pathling requests this information at execution time using the FHIR Terminology Service API.

#### Subsumption testing

Code subsumption testing is particularly useful for terminologies such as SNOMED CT, which feature deep hierarchies and large numbers of concepts. The functions “subsumes” and “subsumedBy” allow us to efficiently move up and down the subsumption hierarchy within our queries, based on the ontological data held by the terminology server.

An example of an expression that uses subsumption testing follows:
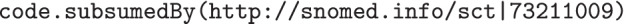


This expression could be used to filter Condition resources to only those that are a type of “Diabetes mellitus” (which is the concept referred to by the identifier “73211009”). Within the international release of SNOMED CT (February 2022), there are 119 concepts that would match this query.

#### Concept translation

Concept translation allows us to traverse mappings between codes that are known to the terminology server. The “translate” function takes a set of input concepts and asks the terminology server to return the targets of mapping relationships found within a particular map. This is particularly useful in the area of data analytics, where the harmonization of heterogeneous data capture is a common requirement. It can also be used with maps that are implicitly defined within particular terminologies. One example of this is the use of SNOMED CT historical associations to translate inactive codes to the updated codes that replace them.

Another example translates SNOMED CT codes to Read CTV3:
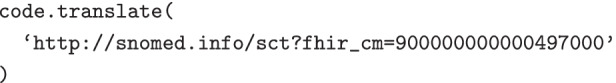


The argument refers to a SNOMED CT implicit ConceptMap URI. This is the standard mechanism within FHIR for describing a simple map within SNOMED CT. This URI refers to a map that is released as part of the international edition of SNOMED CT (identified by “900000000000497000”).

The translate function can also be “reversed”, which instructs it to retrieve the source concepts within a map when given a set of target concepts. It is also capable of filtering results to a defined subset of relationship types, such as “related to”, “broader”, “narrower” and “equivalent”.

#### Property and designation lookup

Property and designation lookup uses a FHIR terminology service to join across from coded data to the known attributes of those codings.

Examples of information that can be retrieved via properties and designations are synonyms, parent and child concepts, display text in other languages and more. Complex terminologies such as SNOMED CT and LOINC also provide ontological information via properties, such as finding sites for disorders, or methods for procedures.

Property and designation lookup has not yet been implemented within Pathling. The details of the design and implementation of this feature will be the subject of a future paper.

### Pathling

**Fig. 5:**
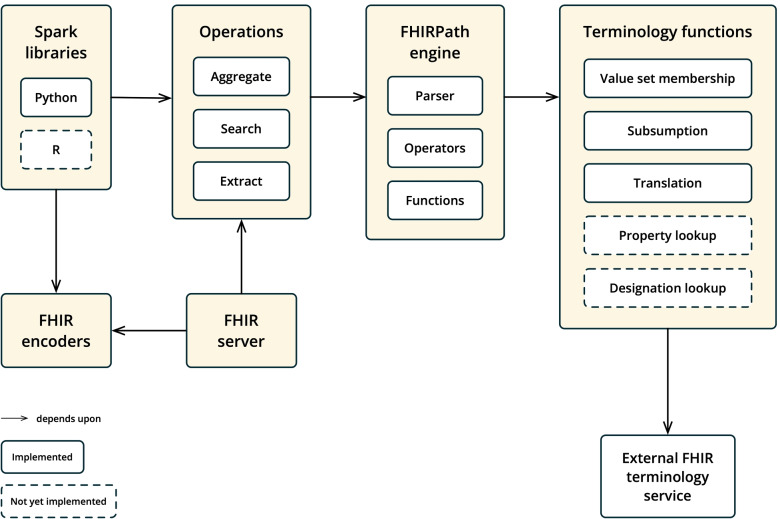
Implementation components

Pathling is an implementation of the FHIR Analytics API concept within this paper, and has been made freely available under a permissive open source license via GitHub. It is written using Java and Scala, and distributed as a Docker image. The components that comprise the Pathling solution are shown in Fig. 5.

Pathling is an Apache Spark application at its core. The work done by Cerner on the Bunsen library was adopted and enhanced to enable the import of FHIR data into Spark. Each FHIR resource is represented as a single Spark data set, utilizing data types such as “struct” and “array” to represent nested data that often has multiple cardinalities and optionality. Each data set is written to a Parquet file for persistence and subsequent retrieval.

A FHIRPath parser, written using ANTLR [26], translates FHIRPath expressions into queries that can be executed using Spark. Parsing of each sub-expression results in an object that represents a FHIRPath expression, along with its associated Spark query representation. These FHIRPath objects can then be used as inputs to various FHIRPath functions and operators that have been implemented as Spark query transformations.

The terminology functions manifest as Spark mapping operations, where input codings are deduplicated and sent to the configured FHIR terminology service. Multiple requests are made in parallel to resolve queries about different portions of the coded data, based upon a configurable level of parallelism. Ontoserver [27] was primarily used for the development of this capability, as it is an existing mature implementation of the FHIR Terminology Services API with good feature coverage and high performance suitable for analytic workloads.

The Aggregate, Search and Extract operations compose individual FHIRPaths into a composite query. HAPI is used as a framework for exposing the operations within an API that can be consumed by standard FHIR client software. The results of each operation query is transformed into the appropriate FHIR resource for return to the user, whether that be a Parameters, OperationOutcome or Bundle resource.

Pathling implements the asynchronous request pattern defined within the FHIR specification to accommodate long-running queries over large data sets. The asynchronous pattern is based upon RFC 7240 [28], and utilizes standard HTTP headers such as “Prefer” and “Content-Location” to provide a mechanism for querying the status of a job and eventually receiving the final result.

The use of Docker as a distribution format enables users to deploy Pathling using their own infrastructure. This makes it easier for computation to take place where the data currently resides, avoiding egress across boundaries of data custodianship.

### Libraries

It is our intent that the components that make up the Pathling server implementation can be used independently of each other and re-composed into other novel solutions. The first step that we have taken towards this is to make the individual modules that make up the server implementation available via Maven Central. This allows them to be used independently within projects that use Java, Scala and other Java Virtual Machine (JVM) languages. For example, the encoders module can be used to encode FHIR data into Spark data frames, which can then be queried via SQL.

We have also made a library available to Python users through the Python Package Index (PyPI). The Pathling Python API provides access to the Spark encoders and the terminology functions directly from Python, without the need for a running Pathling server. This allows for closer integration with Python-based data science workflows, and also the incorporation of Pathling functionality into bespoke Python applications.

We also have plans to implement methods within the Python API that provide access to the Aggregate and Extract operations, which will see the library reach functional parity with the server implementation. A package for the R language is also planned, which will provide equivalent functionality to the Python library for R users.

### User interface

**Fig. 6:**
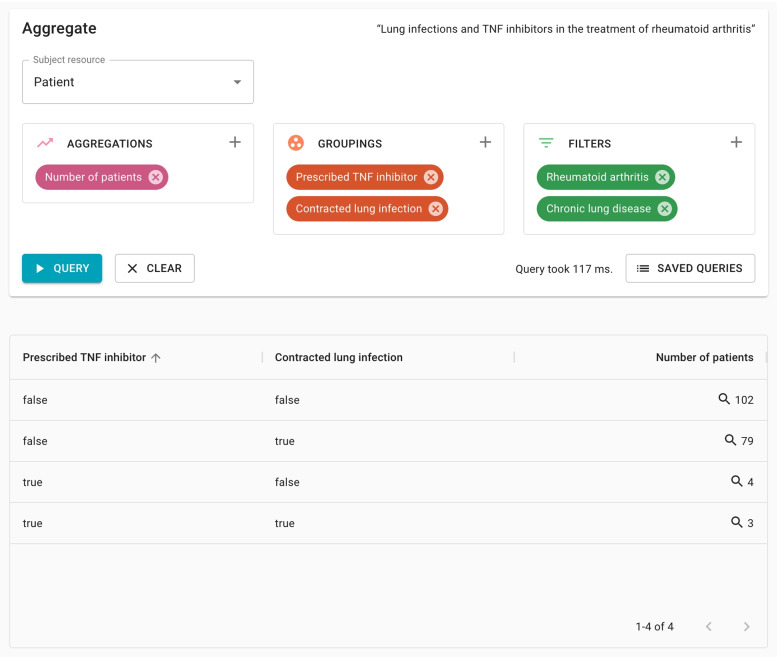
Experimental user interface for exploratory data analysis

As a demonstration of its utility, the Pathling API was used to develop an experimental exploratory data analysis user interface, showing the use of the Aggregate and Search Operations within a generic tool for exploring FHIR data sets (see Fig. 6). A number of synthetic FHIR data sets were created using Synthea [29] that allowed for the demonstration of the features of the API, and the underlying terminology capability.

## Results

### Verification and performance testing

In order to evaluate the correctness of the implementation, a suite of 1,355 automated JUnit tests was developed. This suite includes:

1. A Docker image test, which runs the container and interacts with the FHIR API to import data and run queries;
2. Integration tests, which verify the behavior of the server stack;
3. Query tests, which verify the results from operations such as Aggregate, Search and Extract, and;
4. Unit tests, which verify the behavior of individual classes and methods within the implementation.

In addition to this, we have executed a performance test. To provide the underlying data for this test, we used Synthea to generate three (3) synthetic FHIR data sets in NDJSON format, shown in Table 7. The test was executed on a MacBook Pro (16-inch, 2021) with an M1 Max processor (10 cores) and 32 GB of RAM, using Azul Zulu JDK 11.

**Table 7:** Testing data sets generated with Synthea

Data set #	Number of patients	Total resources	Size on disk (NDJSON)
1	11	7,126	9.8 MB
2	968	739,229	1.1 GB
3	11,516	7,633,526	11.32 GB

The query used within the performance test counts the number of Patients that have an associated pneumonia diagnosis, filtered by those that have an influenza vaccination recorded. It joins from the Patient resource to the Condition resource, and also from the Patient resource to the Immunization resource. This query intentionally does not incorporate terminology functionality, which makes it easier to reason about from a performance perspective.
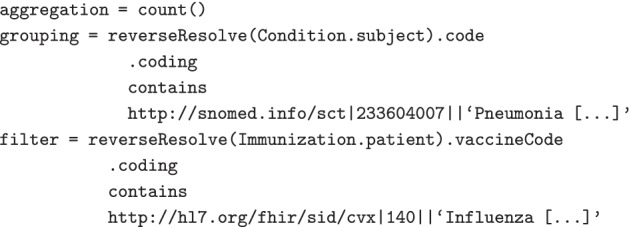


The test was executed against each data set three (3) times, and the mean execution time calculated. The results of the test include cold and warm execution times, shown in Table 8. There is an overhead within the first (cold) execution of a query after startup, where the data in the subject tables are retrieved from storage and loaded into memory. Execution times are measured as the total time from request to response on the client side, which was co-located on the same machine.

**Table 8:** Performance test results

Data set #	Mean cold execution time (ms)	Mean warm execution time (ms)
1	5,164	2,394
2	5,150	2,565
3	7,317	4,725

### Usability

Pathling is being actively used by a number of different users with a range of different levels of domain knowledge and technical ability, and we have had an opportunity to get some feedback on its usability.

While the use of Pathling’s abstractions over FHIR and raw SQL has shown benefits, the effort required in learning FHIRPath is significant. In its current form, Pathling is a tool that makes it easier to build FHIR analytics solutions for data scientists and analysts, rather than being a comprehensive end-to-end solution in its own right.

We plan to invest more effort in user interfaces that leverage the work that has been done within Pathling and the FHIR specification itself. This includes research into visual expression builders for FHIRPath and terminologies like SNOMED CT, which will provide additional tools for developers building data analytics applications on FHIR.

## Discussion

### Comparison of solutions

In order to examine functionality, efficacy and performance, we have created a simple scenario that can be used for targeted comparison across different tools and methods. The scenario is COVID-19 management, specifically the task of ensuring that high-risk patients are vaccinated ahead of major outbreaks.

The scenario comprises two tasks. The first task is to query a synthetic FHIR data set for the number of patients aged between 18 and 60 that have not been vaccinated against COVID-19 and are also at high risk of severe disease, based on their medical history (the “aggregation query”). The second task is to generate a list of unvaccinated, high risk patients and their contact details, to simulate input to a recalls and reminders workflow (the “extraction query”).

The test data that has been used within this comparison is Test Data Set 2, as shown within Table 7.

The data is assumed to be available as FHIR NDJSON, the standard format for interchange of bulk FHIR data. It is also assumed that a FHIR terminology server will be used for the resolution of terminology queries.

We determine whether a patient has been vaccinated against COVID-19 by checking for an associated immunization record with any of the CDC Vaccine Administered (CVX) codes in Table 9. The FHIR value set that represents these codes is shown in Additional File 6.

**Table 9:** COVID-19 vaccine codes *example*

Code	Description
212	COVID-19 vaccine, vector-nr, rS-Ad26, PF, 0.5 mL
207	COVID-19, mRNA, LNP-S, PF, 100 mcg/0.5 mL dose
208	COVID-19, mRNA, LNP-S, PF, 30 mcg/0.3 mL dose

For the purposes of this scenario, patients are determined to be “high risk” if they meet any of the following three conditions:

1. Diagnosed with chronic kidney disease;
2. Diagnosed with heart disease, or;
3. Recorded body mass index (BMI) greater than 30.

Chronic kidney disease is defined as any SNOMED CT concept that is subsumed by “Chronic kidney disease” (which is the concept identified by “709044004”).

Heart disease is defined as any SNOMED CT concept that is a type of “Cardiovascular disease” with a finding site of “Structure of heart”. This is formally described by the following ECL expression:
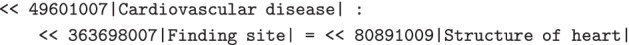


Finally, BMI greater than 30 is determined by the presence of an Observation resource with a LOINC code of “Body mass index (BMI) [Ratio]” (identified by “39156-5”) that also has a Quantity value with a UCUM code of “kg/m2” and a value greater than 30.

For simplicity, we assume that the time of the immunization, diagnosis or observation is not important in this scenario.

We will first demonstrate how Pathling can be used to solve the stated problem, and then move on to a comparative analysis of other solutions.

#### Pathling server

First we will examine the process for performing the necessary analysis using a running instance of the Pathling FHIR server, specifically its Import, Aggregate and Extract operations.

We used the Import operation to load the FHIR NDJSON files and prepare them for subsequent query. A file URL was provided for each of the Patient, Immunization, Condition and Observation resources, instructing the Pathling server to read them from the local file system and encode them into Parquet tables at the configured warehouse location (also on the local file system).

We then used the “aggregate” operation to calculate the number of Patient resources, grouped by vaccination status and high risk, and filtered to the 18-60 age group. The parameters used with the Aggregate operation are shown in Additional File 1.

A tabular representation of the result of the query is shown in Table 10.

**Table 10:** High risk unvaccinated patients - aggregate query result

Vaccinated against COVID-19	High risk	Number of patients
true	false	266
true	true	215
false	false	81
false	true	85

Finally, we used the “extract” operation to list the patients that were both unvaccinated against COVID-19 and also high risk, along with their risk factors, names and contact details. The parameters used with the Extract operation are shown in Additional File 2.

This returns a link to a downloadable CSV file, five rows of which are reflected in Table 11.

**Table 11:** High risk unvaccinated patients - extract query result

Family name	Given name	Phone number	Chronic kidney disease	Heart disease	BMI>30
Abernathy524	Kathline630	555-746-7353	true	false	true
Bartell116	Rhett759	555-257-6514	false	false	true
Bashirian201	Aldo414	555-300-9051	false	false	true
Beahan375	Neva514	555-809-1747	false	false	true
Bednar518	Chase54	555-812-1196	false	false	true

A Jupyter Notebook describing the Pathling server workflow in full can be found in Additional File 3.

#### Pathling Python API

The same result can be derived through the use of the Pathling Python API, a method that does not have the requirement to interact with a running server. The Python API is a package available from PyPI, and the only dependencies are Python itself and a Java runtime environment.

The Python API provides access to the encoders within Pathling, which allow for FHIR data to be converted into Spark data frames. The work of navigating and transforming the FHIR data structures is largely manual, as we have not yet implemented equivalent abstractions for the Aggregate and Extract operations within Python.

The Python API has a set of terminology functions which automate the task of resolving terminology queries over Spark data frames. In this scenario, we call upon the ‘member_of‘ and ‘subsumes‘ functions to assist with creating columns that represent the risk factors of chronic kidney disease and heart disease.

The Python API removes the requirement to persist the data to Parquet or any other type of storage, as the data frames can be operated upon in memory. This makes it ideal for use within data science workflows and data transformation pipelines. It can also be used along with the streaming functionality within Spark to consume data from real-time sources and process it continuously.

A Jupyter Notebook describing the Python API workflow in full can be found in Additional File 4.

#### Python and Pandas

For comparison we also performed the necessary analysis using only the Python programming language, modules from the Python standard library and the Pandas data analysis package.

We loaded the FHIR NDJSON data into Python dictionary structures using the “json” package within the Python standard library. We then selected only the data necessary for the analysis and loaded that into a set of Pandas data frames.

The Pandas data frames were then supplemented with additional columns containing the results of the necessary terminology queries, e.g. “Chronic kidney disease”, “Heart disease”. Requests were made to the “validate-code” and “subsumes” operations of a FHIR terminology server using the Python HTTP library. Simple memoization was used to deduplicate the requests sent to the terminology service, simulating a client-side cache.

Once the construction of the data frames was complete, they were joined together using the patient identifiers. Filtering the cohort on age was achieved using the date parsing and subsetting operations within Pandas.

Implementation of a pure Python solution to this problem requires a higher level of knowledge of FHIR and the terminology API, and involves a larger volume of code to be written. Also, this implementation is tailored to this particular problem, makes numerous assumptions about the structure and content of the data and would not generalize well to other FHIR analysis use cases. It does not handle error states, missing data and other edge cases.

A Jupyter Notebook describing the pure Python workflow can be found in Additional File 5.

#### Clinical Quality Language (CQL)

The CQL specification [30] provides a standard representation of clinical quality logic that can be used to query and calculate quality measures over health data sets, including those modeled using FHIR.

It would be possible to create a solution to the stated problem using a CQL-enabled FHIR server, integrated with terminology services. The full implementation of this solution is outside of the scope of this paper, but we can discuss some of the different features of CQL that can be leveraged towards this type of problem.

CQL can be used to perform the “aggregate query” part of this problem using two main artifacts: the Library resource and the Measure resource. The Library resource is used to store clinical quality logic, such as the definitions of vaccination status and risk factors. The Measure resource is able to refer to the definitions within one or more Library resources and expose them as calculations.

The “evaluate-measure” operation could then be executed to retrieve a MeasureReport resource containing a similar result to the result of the Pathling Aggregate operation.

One assumption here is that the CQL implementation would have the ability to either resolve the required terminology queries itself, or delegate this work to an external FHIR terminology server with the necessary capabilities.

CQL also has the ability to execute “subject-list” and “subject” reports, which could be used to construct the list of unvaccinated, high risk patients along with their risk factors and contact details.

Another option could be to integrate a “CQL engine” more tightly into a data science workflow or processing pipeline, obviating the need for a standing CQL-enabled FHIR server.

There is overlap between the ideas within CQL and the work in this paper, but the focus and intended use are quite different. CQL focuses upon providing a standard mechanism for creating shareable definitions of cohorts, measures and indicators, while Pathling has an emphasis on data transformation, exploratory data analysis, and the provision of an API that enables applications to more easily deliver analytic query capabilities.

#### FHIR Search API

The FHIR specification contains a Search API, which takes a set of parameters that describe how to filter the collection of resources held within a server.

Applied to the stated problem, it would be possible to use the FHIR Search API to get counts of patients with particular risk factors, or vaccination status. For example, the HTTP request below would retrieve all patients with an associated chronic kidney disease condition:
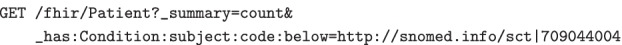


However, it starts to get difficult to combine all of the necessary conditions, as the FHIR Search API only supports simple combinations of Boolean expressions. It is not possible, for example, to combine three conditions on two different search parameters using OR logic, and then combine them with a fourth condition using AND logic.

In order to fully implement a solution to this problem using only the core FHIR API, it would be necessary to query data in bulk out of the server and do some processing, filtering and transformation within client code. This exercise is outside the scope of this paper.

Pathling server has its own FHIR Search API, but in place of the standard API it provides a “fhirPath” search profile. This search profile allows the user to express search criteria using the FHIRPath language. FHIRPath provides additional expressive power relative to the FHIR Search API, such as:

1. The ability to refer to any element, not just those that are the subject of a defined search parameter;
2. Unlimited nesting and bracketing of expressions;
3. The ability to follow resource references that traverse multiple levels of relationships, and;
4. Support for complex terminology operations within criteria.

#### PhenoMan

PhenoMan is a client application that can be used to specify phenotypes, calculate their values and write them back into a FHIR server in the form of Observation resources [31]. These Observation resources can then be queried alongside the other resources within the server using the FHIR Search API. PhenoMan can also be used to model patient cohort selection criteria and convert them into queries that can be executed by the FHIR Search API [32].

Pathling and PhenoMan are largely complementary solutions, and could even be used together with some modification. In some ways PhenoMan has more of an overlap with the functionality of a FHIR terminology service, and one option for integrating with it could be to implement a FHIR terminology service interface that provides access to the ontological knowledge that PhenoMan provides.

Applied to this problem, Phenoman could be used to create derived Observation resources within a FHIR server to represent vaccination status and risk factors. The presence of these Observation resources could then be queried using the FHIR Search API to derive the aggregate results and patient list. The full implementation of this solution is outside the scope of this paper.

#### fhircrackr

A package for the R language called “fhircrackr” is available, which automates the task of downloading resources from a FHIR server and transforming them into R data frames. It delivers similar functionality to what the Extract operation could provide if delivered into an R environment.

This package could assist with the work of downloading and transforming the FHIR source data required for this analysis problem. Aggregation of the data would need to be performed manually in a similar way to the pure Python solution, and terminology queries would also not be assisted by this tool. A full implementation of this solution using “fhircrackr” is outside the scope of this paper.

#### Structured Query Language (SQL)

The final alternative to mention is the use of tools for loading FHIR data into a SQL database. A3 [18] is a project that has taken the approach of providing tools to transform a FHIR data set ahead of time, with the goal of making it easier to query using standard SQL tools and generalized analytic platforms like Google BigQuery.

A3 is capable of normalizing a range of FHIR data structures such as resource references, extensions, dates and recursive data structures to reduce the complexity of the SQL queries required to perform common analytic tasks. The team has also done a lot of work relating to the development of user interfaces for building SQL queries, including the composition of patient cohort selection criteria.

Applied to the stated problem, SQL has the ability to perform the necessary aggregation and extraction operations. However, it would not have built-in support for interacting with the FHIR terminology API, and orchestrating these queries and making their results available would require additional components. A full implementation of this solution involving a load of FHIR data into a service like BigQuery is outside the scope of this paper.

#### Performance comparison

A performance comparison of the three comparable, implemented solutions is summarized in Table 12.

**Table 12:** Performance comparison

Description	Total execution time (s)
Pathling server	90.490
Pathling Python API	27.769
Python and Pandas	61.606

The comparison was executed on a MacBook Pro (16-inch, 2021) with an M1 Max processor (10 cores) and 32 GB of RAM, using Azul Zulu JDK 11.

The Pathling server implementation is at a bit of a disadvantage in this comparison, as it is the only solution that persists the data. The import of the data took 32.009 seconds - the subtraction of which brings its performance back to a similar level to the pure Python implementation.

### Discussion of storage and processing options

The use of Spark and Parquet differentiates Pathling from other FHIR server implementations, which use relational database management systems (RDBMS) [33], NoSQL databases [34] or graph databases [35, 36] as their storage layer. The primary advantages of using Spark are:

1. The ability to apply arbitrary computation within a query, including calls out to external services (e.g. a FHIR terminology server);
2. The ability to scale query processing beyond an individual instance, enabling processing of data sets of unbounded scale, and;
3. Strong support for integrating with the Python and R programming languages, which are widely used in the area of data analytics.

Stream processing support is another advantage that comes with using Spark, although this benefit is yet to be realized within the implementation (see Future work).

These features come at the cost of some increased latency at small data sizes. Another disadvantage is that although Spark is mature and achieving rapid adoption, it remains less familiar to prospective adopters than some of the older technologies.

### Case study

Pathling was applied within a project related to the analysis of data collected within a number of genetic clinics. Diagnoses were collected at the referral (GP or specialist), pre-investigation (initial consultation at clinic) and post-investigation (analysis of test results) stages of the process, and were coded using SNOMED CT.

Pathling was used to perform the following queries over the data:

1. Diagnostic yield by disease grouping, age range and technology employed, and;
2. Count of diagnoses according to whether they improved the specificity of the diagnosis, relative to previous diagnoses.

**Fig. 7:**
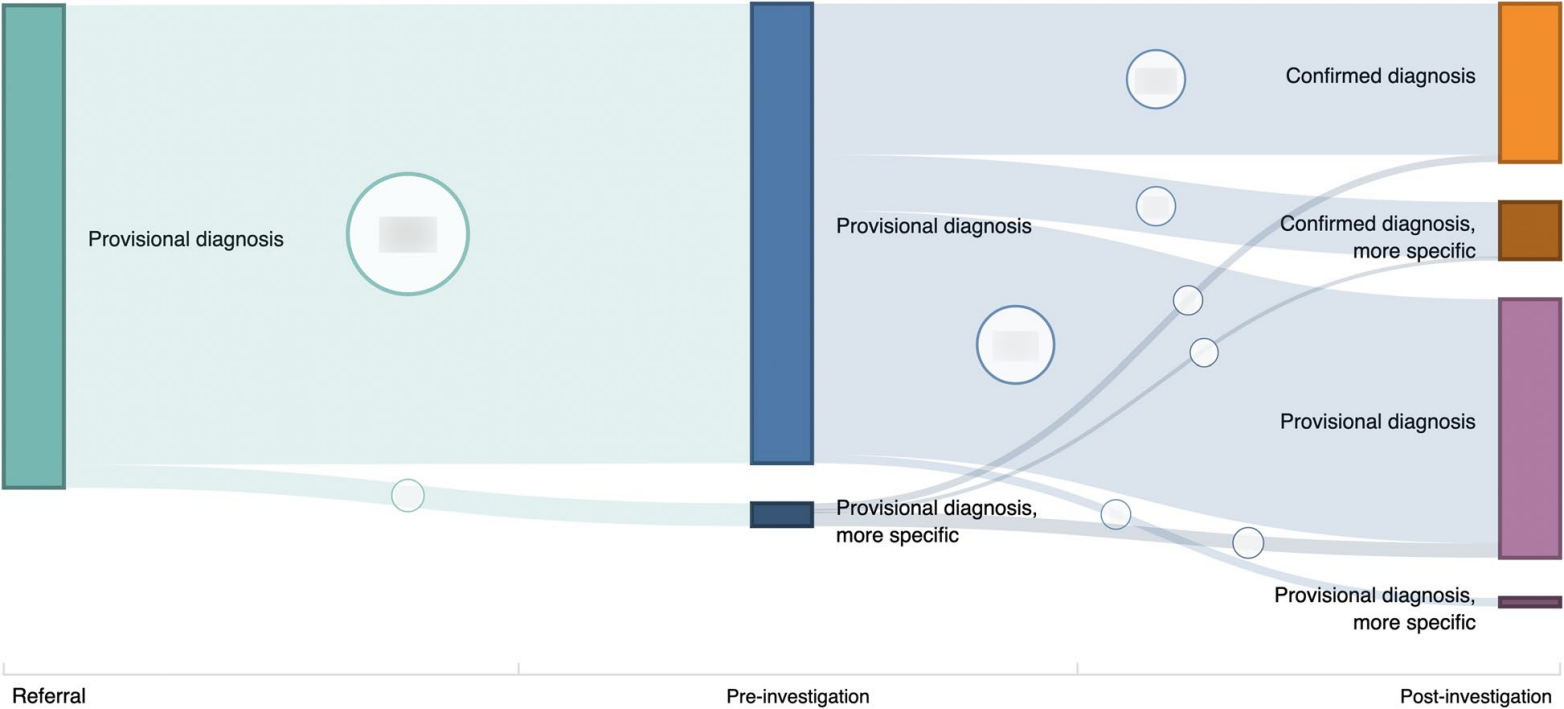
Number of genomic test patients by encounter type, diagnosis verification status and diagnosis specificity relative to previous diagnoses (counts redacted)

The latter query involved the interrogation of the SNOMED CT hierarchy using the “memberOf” function and ECL. A Sankey diagram (see Fig. 7) was combined with a response from the Aggregate Operation to provide a visualization of the results.

The analytic users on this project had three primary goals: (1) calculation of the correct results; (2) use of a process that is efficient, to support many different iterations of the analysis during exploration of the research question, and; (3) use of a process that is repeatable, so that results can be reproduced and similar analyses can later be conducted on other data sets. The use of Pathling allowed the users to meet all three of these goals by removing the need to write bespoke code to transform and pre-process the FHIR data and query SNOMED CT.

### Future work

Further work on new user interfaces will be conducted, specifically in the area of visual expression builders for FHIRPath and SNOMED CT ECL. We are also investigating how Pathling can be used to augment tasks within clinical terminology authoring workflows by allowing FHIR ValueSet authors to query the coverage and frequency of codes within a target data set.

The integration of Pathling functionality within Python and R will be further developed, making not only the encoders available but also the operations, terminology functions and other helper functions for dealing with FHIR.

Pathling will be enhanced to make it possible for it to act as a FHIR Subscription server, pushing changes to its own data to subscribers. The combination of real-time updates and push messaging relating to changes within the data (using criteria described using FHIRPath expressions) could enable the FHIR Analytics API to be used as an engine for driving sophisticated alert systems within the clinical setting.

We also have plans to continue to apply the technology within a number of upcoming partner projects, related to:

1. Powering patient cohort selection for genomic health research;
2. Analysis of SNOMED CT and ICD coded emergency department data relating to trauma;
3. Studying the disease burden of type 2 diabetes upon First Nations People of Australia, developing applications designed to communicate results back to participating communities.

## Conclusions

There is a need for improved tooling in the area of health data analytics, to reduce the skills burden and to improve the consistency and quality of analyses. Our research has identified exploratory data analysis, patient cohort selection and data preparation as promising areas that would benefit from additional investment in tools.

Functions designed to assist analytic users and software developers in these areas have been encapsulated within a server that exposes a FHIR Analytics API. Aggregate, extract and search operations, used in conjunction with FHIRPath expressions and the FHIR Terminology Service, show promise as a way of abstracting away some of the complexity of the underlying data structures while preserving query expressivity.

A standards-based REST API for analytics enables and encourages the establishment of an ecosystem of applications that can be developed rapidly and be used to explore requirements in this rapidly evolving area. It also incentivizes the use of standards within data acquisition and transformation efforts within healthcare organizations.

## Availability and requirements

**Project name:** Pathling

**Project home page:** https://pathling.csiro.au

**Operating system(s):** Java Virtual Machine, platform independent

**Programming language:** Java, Scala, Python

**Other requirements:** Java 11 or higher, Python 3.7 or higher

**License:** CSIRO Open Source Software Licence Agreement

**Any restrictions to use by non-academics:** None

## Supplementary information

**Additional file 1.** Parameters used with the Aggregate Operation as part of the comparison of solutions.

**Additional file 2.** Parameters used with the Extract Operation as part of the comparison of solutions.

**Additional file 3.** Jupyter Notebook showing a comparable analysis process using a Pathling server.

**Additional file 4.** Jupyter Notebook showing a comparable analysis process using the Pathling Python API.

**Additional file 5.** Jupyter Notebook showing a comparable analysis process using Python, the Python standard library and the Pandas data analysis package.

**Additional file 6** FHIR value set representing COVID-19 vaccine codes.

## Data Availability

The source code for v5.4.0 of Pathling, including the definition of the executed test suite, is available at https://doi.org/10.25919/q2vd-a532. A test execution report for v5.4.0 of Pathling is available at: https://github.com/aehrc/pathling/actions/runs/2726818301. The data sets used for performance testing and the comparison of solutions are available at https://doi.org/10.25919/78k0-py81. A Conda environment lock file for reproducing the Jupyter Notebook execution is available at https://doi.org/10.25919/78k0-py81.

## Abbreviations

API: Application Programming Interface
BMI: Body Mass Index
CDC: Centers for Disease Control and Prevention
CDM: Common Data Model
CQL: Clinical Quality Language
CSV: Comma Separated Values
CVX: CDC Vaccine Administered
ECL: Expression Constraint Language
EDA: Exploratory Data Analysis
EHR: Electronic Health Record
FHIR: Fast Healthcare Interoperability Resources
HDFS: Hadoop File System
HTTP: Hypertext Transfer Protocol
ICD: International Statistical Classification of Diseases and Related Health Problems
LOINC: Logical Observation Identifiers Names and Codes
JVM: Java Virtual Machine
NDJSON: Newline Delimited JSON
OHDSI: Observational Health Data Sciences and Informatics
OMOP: Observational Medical Outcomes Partnership
PyPI: Python Package Index
RDBMS: Relational Database Management System
REST: Representational State Transfer
SNOMED CT: SNOMED Clinical Terms
SQL: Structured Query Language
UCUM: Unified Code for Units of Measure
URI: Universal Resource Identifier
URL: Universal Resource Locator

## References

[1] Braunstein ML. Health Informatics on FHIR: How HL7's New API is Transforming Healthcare. Cham: Springer International Publishing; 2018.

[2] Lehne M, Luijten S, Imbusch PVFG, Thun S, et al. The Use of FHIR in Digital Health-A Review of the Scientific Literature. Maastricht: GMDS; 2019. pp. 52–58. 10.3233/SHTI190805 31483254

[3] Giannangelo K, Fenton SH. SNOMED CT survey: an assessment of implementation in EMR/EHR applications. Perspect Health Inf Manag. 2008;5:7. PMC2396499 18509501

[4] Tukey JW (1962). The future of data analysis. Ann Math Stat.

[5] SNOMED International. Expression Constraint Language - Specification and Guide. 2016. http://snomed.org/ecl. Accessed 10 Aug 2022.

[6] Kenrick N, Svabova L, Nica E (2019). Real-time health-related data, wearable medical sensor devices, and smart cyber-physical systems. Am J Med Res.

[7] Hripcsak G, Duke JD, Shah NH, Reich CG, Huser V, Schuemie MJ (2015). Observational Health Data Sciences and Informatics (OHDSI): opportunities for observational researchers. Stud Health Technol Inform.

[8] Observational Health Data Sciences and Informatics. ATLAS - A unified interface for the OHDSI tools. 2018. https://www.ohdsi.org/atlas-a-unified-interface-for-the-ohdsi-tools/. Accessed 10 Aug 2022.

[9] Ma C, Frankel H, Beale T, Heard S (2007). EHR query language (EQL)-a query language for archetype-based health records. Medinfo..

[10] The openEHR Foundation. OpenEHR - Archetype Query Language (AQL). 2008. https://specifications.openehr.org/releases/QUERY/latest/AQL.html. Accessed 10 Aug 2022.

[11] Liu D, Sahu R, Ignatov V, Gottlieb D, Mandl KD. High performance computing on flat FHIR files created with the new SMART/HL7 Bulk Data Access Standard. In: AMIA Annual Symposium Proceedings, vol. 2019. Bethesda: American Medical Informatics Association; 2019. p. 592. PMC7153160 32308853

[12] Apache Software Foundation. Apache Parquet; 2015. https://parquet.apache.org/. Accessed 10 Aug 2022.

[13] Cerner Innovation. cerner/bunsen: Explore, transform, and analyze FHIR data with Apache Spark. 2017. https://github.com/cerner/bunsen. Accessed 10 Aug 2022.

[14] Zaharia M, Chowdhury M, Franklin MJ, Shenker S, Stoica I (2010). Spark: Cluster computing with working sets. HotCloud..

[15] Ryan Brush. FHIR/sql-on-fhir: SQL on FHIR Proposal. 2018. https://github.com/FHIR/sql-on-fhir. Accessed 10 Aug 2022.

[16] Google. Protocol Buffers | Google Developers. 2012. https://developers.google.com/protocol-buffers/. Accessed 10 Aug 2022.

[17] Google. google/fhir: FHIR Protocol Buffers. 2018. https://github.com/google/fhir. Accessed 10 Aug 2022.

[18] Daniel Gottlieb and Joshua Mandel. A3 - Prototype of FHIR-native ETL+Q Platform. 2020. https://github.com/sync-for-science/a3. Accessed 10 Aug 2022.

[19] Mandl KD, Gottlieb D, Mandel JC, Ignatov V, Sayeed R, Grieve G (2020). Push Button Population Health: The SMART/HL7 FHIR Bulk Data Access Application Programming Interface. NPJ Digit Med.

[20] Jones J, Gottlieb D, Mandel JC, Ignatov V, Ellis A, Kubick W, et al. A landscape survey of planned SMART/HL7 bulk FHIR data access API implementations and tools. J Am Med Inform Assoc. 2021;28(6):1284-87. 10.1093/jamia/ocab028 PMC8661398 33675659

[21] Computational Health Informatics Program, Boston Children’s Hospital. Draft Bulk Import Implementation Guide. 2019. https://github.com/smart-on-fhir/bulk-import/blob/master/import.md. Accessed 10 Aug 2022.

[22] Shvachko K, Kuang H, Radia S, Chansler R. The hadoop distributed file system. In: 2010 IEEE 26th symposium on mass storage systems and technologies (MSST). Incline Village: IEEE; 2010. p. 1–10.

[23] Thorsten Hoeger, Chris Dew, Finn Pauls and Jim Wilson. NDJSON. 2013. https://github.com/ndjson/ndjson-spec. Accessed 10 Aug 2022.

[24] Alexander M, Jelen B. Pivot table data crunching. Indianapolis: Pearson Education; 2001.

[25] Health Level Seven International. FHIRPath. 2020. http://hl7.org/fhirpath/. Accessed 10 Aug 2022.

[26] Parr TJ, Quong RW (1995). ANTLR: A predicated-LL (k) parser generator. Softw Pract Experience.

[27] Metke-Jimenez A, Steel J, Hansen D, Lawley M (2018). Ontoserver: a syndicated terminology server. J Biomed Semant.

[28] Internet Engineering Task Force (IETF). Prefer Header for HTTP. 2014. https://datatracker.ietf.org/doc/html/rfc7240#section-4.1. Accessed 10 Aug 2022.

[29] Walonoski J, Kramer M, Nichols J, Quina A, Moesel C, Hall D (2018). Synthea: An approach, method, and software mechanism for generating synthetic patients and the synthetic electronic health care record. J Am Med Inform Assoc.

[30] Health Level Seven International. Clinical Quality Language (CQL). 2014. https://cql.hl7.org/. Accessed 10 Aug 2022.

[31] Uciteli A, Beger C, Kirsten T, Meineke FA, Herre H (2020). Ontological representation, classification and data-driven computing of phenotypes. J Biomed Semant.

[32] Uciteli A, Beger C, Wagner J, Kirsten T, Meineke FA, Stäubert S, et al. Ontological modelling and FHIR Search based representation of basic eligibility criteria. GMS Med Informatik Biom Epidemiol. 2021;17(2). https://www.egms.de/static/en/journals/mibe/2021-17/mibe000219.shtml.

[33] University Health Network. HAPI FHIR. 2014. https://github.com/hapifhir/hapi-fhir. Accessed 10 Aug 2022.

[34] Asymmetrik. Asymmetrik FHIR API Server. 2017. https://github.com/Asymmetrik/node-fhir-server-core. Accessed 10 Aug 2022.

[35] Optum. CyFHIR. 2020. https://github.com/Optum/CyFHIR. Accessed 10 Aug 2022.

[36] Fette G, Kaspar M, Liman L, Ertl M, Krebs J, Störk S, et al. Implementation of a HL7-CQL Engine Using the Graph Database Neo4J. In: GMDS. Dortmund: German Association of Medical Informatics, Biometry and Epidemiology; 2019. p. 46–51. 10.3233/SHTI190804 31483253

